# Inter- and intrabreed diversity of the major histocompatibility complex (MHC) in primitive and draft horse breeds

**DOI:** 10.1371/journal.pone.0228658

**Published:** 2020-02-03

**Authors:** Joanna Jaworska, Katarzyna Ropka-Molik, Izabela Wocławek-Potocka, Marta Siemieniuch

**Affiliations:** 1 Department of Gamete and Embryo Biology, Institute of Animal Reproduction and Food Research, Polish Academy of Sciences, Olsztyn, Poland; 2 Department of Animal Molecular Biology, National Research Institute of Animal Production, Balice, Poland; 3 Research Station of the Institute of Reproduction and Food Research, Polish Academy of Sciences in Popielno, Ruciane-Nida, Poland; University of Minnesota College of Veterinary Medicine, UNITED STATES

## Abstract

**Background:**

Polymorphism of major histocompatibility complex (MHC) genes ensures effective immune responses against a wide array of pathogens. However, artificial selection, as performed in the case of domestic animals, may influence MHC diversity. Here, we investigate and compare the MHC diversity of three populations of horses, for which different breeding policies were applied, to evaluate the impact of artificial selection and the environment on MHC polymorphism.

**Methods:**

Samples of DNA were taken from 100 Polish draft horses, 38 stabled Konik Polski horses and 32 semiferal Konik Polski horses. MHC alleles and haplotype diversity within and between these populations of horses was estimated from 11 MHC microsatellite loci.

**Results:**

MHC diversity measured based on allelic richness, observed heterozygosity, expected heterozygosity and polymorphism content was similar across the MHC microsatellite loci in all three populations. The highest expected heterozygosity was detected in semiferal primitive horses (He = 0.74), while the lowest was calculated for draft horses (He = 0.65). In total, 203 haplotypes were determined (111 in Polish draft horses, 43 in semiferal Konik Polski horses and 49 in stabled Konik Polski horses), and four haplotypes were shared between the two populations of Koniks. None of these haplotypes were present in any of the previously investigated horse breeds. Intra-MHC recombination events were detected in all three populations. However, the population of semiferal Konik horses showed the highest recombination frequency among the three horse populations. In addition, three recombination events were detected.

**Conclusions:**

These results showed that despite the different breeding policies, the MHC allele and haplotype diversity was similarly high in all three horse populations. Nevertheless, the proportion of new haplotypes in the offspring was the highest in semiferal Konik Polski horses, which indicates the influence of the environment on MHC diversity in horses. Thus, we speculate that the genetic makeup of the domestic horse MHC might be more strongly influenced by the environment than by artificial selection. Moreover, intra-MHC conversion, insertion, and deletion and intra-MHC recombination may be proposed as mechanisms underlying the generation of new MHC haplotypes in horses.

## Introduction

The major histocompatibility complex (MHC) genes play an important role in innate and adaptive immune responses [1]. Consequently, MHC may affect the development of autoimmune diseases and susceptibility or resistance to pathogens [1–3]. In addition to its role in immune defense, MHC is involved in reproductive processes such as placental development and fetomaternal tolerance [4, 5]. It has been established that MHC genes are the most polymorphic genes within the vertebrate genome [1]. In wild species, high polymorphism is maintained by balancing selection, which may occur via two mechanisms [6–9]. First, MHC heterozygotes are believed to be preferred over MHC homozygotes because they are able to present a wider range of antigens [2, 3, 10]. Second, specific MHC alleles that ensure protection against pathogens present currently in the environment are favored [3, 11, 12]. In this case, the preference for MHC changes over time together with the change in pathogen composition. In contrast, domestic animals undergo artificial selection [13, 14]. Thus, in addition to pathogen/fitness-related pressure, the pool of MHC genes present in domestic animals may be influenced by a human preference for specific traits [6, 13, 14].

Polish draft horses and Konik Polski horses are among the breeds that have never been characterized with regard to MHC diversity. Draft horses were introduced to Poland in the first half of the 19^th^ century [15]. From the beginning, the breed was continuously improved by cross-breeding with other draft breeds, such as French and Swedish Ardennes, Bretons, Belgians or Percherons [15, 16]. Interestingly, draft horses, including Polish draft horses, suffer from a high incidence of retained fetal membranes (RFMs), which is suggested to be caused by the similarity between fetal and maternal MHC [17, 18].

Konik Polski horses are a primitive breed that originated directly from the Tarpan [19, 20]. The last representatives of this species were domesticated in the early 19^th^ century, and then, Tarpans were crossbred with domestic horses, including draft horses [19–21]. However, in Poland, the conservation program for Tarpan descendants has been ongoing since 1936, and Konik Polski horses are currently under a genetic resource protection program according to the Convention on Biological Diversity (https://www.cbd.int/). The studbook for Konik Polski horses was closed in 1985, and no admixture of other breeds was allowed. Konik Polski horses are characterized by high fertility, longevity, good use of low-quality forage and adaptability to harsh conditions. Because of the qualities of these horses, two types of maintenance are allowed: conventional stable-based maintenance and semiferal maintenance, with the least possible human influence, which means that any veterinary procedures such as deworming are not allowed [19, 22, 23]. Clearly, the type of selection as well as living conditions used for both breeds differs; Konik Polski horses are kept pure, while Polish draft horses are continuously improved by addition of other draft breeds of horses.

The available literature shows that continuous selection may influence not only the phenotypic traits of modern horses but also their genomes, including MHC genes. Increased susceptibility to immune-based diseases or viral infections has been reported in some breeds but not in others or is associated with specific MHC alleles present in an individual [17, 18, 24–28]. Nevertheless, the number of horse breeds enrolled in studies of MHC is limited.

Intra-MHC microsatellites are well established tools for characterizing equine MHC [29–35] as well as for finding associations between specific MHC alleles and disease susceptibility [24, 25, 28]. In addition, MHC microsatellites can detect differences within and between MHCs of different horse breeds [33]. Here, we used a panel of MHC microsatellites that correspond to MHC haplotypes defined previously by serological methods. Moreover, these microsatellites were used in other horse breeds. The aim of the study was to investigate the polymorphism of MHC within and between two breeds of horses, kept either in feral or domestic conditions: Konik Polski and Polish draft horses. We wanted to assess the MHC diversity by determining the polymorphism of MHC microsatellites and MHC haplotypes. Use of the same MHC microsatellites will allow comparison of the results with those for previously tested horse populations.

## Methods

### Ethical note

Blood samples from horses were taken during annual parentage testing required by studbook regulations. No experimentation was performed in view of the European Directive 2010/63/EU and the Polish laws related to ethics in animal experimentation. According to the European Directive 2010/63/EU on the protection of animals used for scientific purposes (chapter 1, article 1.5), “practices undertaken for the primary purpose of identification of an animal” do not need the approval of the Institutional Animal Care and Use Committee, which was confirmed by the Local Ethical Committee (LKE.065.07.2019). Owners of the animals provided consent and agreed to the use of blood samples.

### Animals

The analyzed horse population represented 100 Polish draft horses, including 49 mares, 43 offspring and eight stallions from one stud, which was founded in 1952 and located in the northeastern part of Poland (Population 1, Pop1). The Konik Polski horses came from one stud, which was founded in 1949 and was from the same region of the country. From the moment of stud foundation, Konik Polski horses from this stud were not crossbred with other breeds. In 1955, two maintenance systems were applied: feral and stabled. Due to the two different maintenance systems, horses were divided into two populations: 32 horses, which included 16 mares, 12 offspring, and four stallions, represented population 2 (Population 2, Pop2), which was considered to consist of semiferal Konik Polski horses. The animals live in a 16.2 km^2^ sanctuary and breed freely. However, due to the limited area, almost all offspring are removed every year. If necessary, young males and females are left for parent replacement. The second population of Konik Polski horses (Population 3, Pop3) was represented by 38 horses, including 19 mares, 15 offspring, and four stallions. These horses were kept and bred under conventional stabled conditions. The two populations of Konik Polski horses were not crossbred since 1965; thus, they were treated as separate populations.

### Genetic analyzes

#### DNA isolation

Blood was taken from the jugular vein into 8.5-ml ACD tubes (with 1.5 mL of solution A, consisting of trisodium citrate, 22.0 g/L; citric acid, 8.0 g/L; and dextrose 24.5 g/L)

Peripheral blood lymphocytes were isolated with RBC lysis solution (Qiagen, Hilden, Germany, #158902). After centrifugation, 1 mL of the buffy coat was transferred to 3 ml of RBC lysis solution and incubated for 5 min Then, the tube was centrifuged at 470 x g for 5 minutes, and the supernatant was removed. The lymphocyte pellet was washed with PBS three times, and after the last wash, it was transferred to a 1.5-mL Eppendorf tube. The supernatant was removed, and the lymphocytes were snap-frozen in liquid nitrogen, transferred to an ultrafreezer at -80°C and stored in these conditions until DNA isolation.

The DNeasy Blood & Tissue Kit (Qiagen, Hilden, Germany, #69506) was used to isolate genomic DNA according to the manufacturer’s protocol. Isolated DNA was stored at -20°C until further analysis.

#### MHC microsatellite typing

Eleven equine MHC microsatellites were amplified in three multiplex PCRs (S1 Table). Each reaction contained 2 μl of genomic DNA, 6.25 μl of DreamTaq PCR Master Mix (2X) (ThermoScientific, Waltham, Massachusetts, USA, #K1072), 0.2 μl of fluorescently labeled forward and reverse primers and ddH_2_O to a total volume of 14.5 μl per well. The following conditions were used for the PCRs: 95°C for 3 min, followed by 35 cycles of 95°C for 30 s, 60°C for 30 s, and 72°C for 60 s and 72°C for 10 min for the final extension. Electrophoresis on a 3% agarose gel with ethidium bromide confirmed the specificity of MHC microsatellite amplification.

To analyze the DNA fragments, 1 μl of every PCR product was mixed with 14 μl of Hi-Di^™^ formamide (Applied Biosystems®, Foster City, California, USA, #4311320) and 0.5 μl of GeneScan^™^ 500 LIZ^™^ Size Standard (Applied Biosystems®, Foster City, California, USA, #4322682) to a final volume of 15.5 μl on a 96-well plate. Next, the PCR products were denatured at 95°C for 5 min and placed instantly on ice. Fragments were then separated and sized on a 3500xL Genetic Analyzer capillary sequencer (Applied Biosystems®, Foster City, California, USA). GeneMapper TM 4.0 software (Applied Biosystems®, Foster City, California, USA) was used for fragment length analysis.

#### MHC haplotype phasing

This experiment was performed according to Holmes et al. [36]. Briefly, related horses in each population were used for identification of MHC haplotypes. The MHC alleles present in trios of dams, sires and offspring or parent-offspring pairs were compared. This facilitated the determination of parental alleles in the offspring but not in the parents. Visual inspection and comparison of MHC haplotypes between related horses allowed us to explore possible genetic events, i.e., recombination, conversion, insertion or deletion, which may lead to the generation of new MHC haplotypes in offspring.

### Statistical analysis

#### MHC microsatellites

The number of observed alleles per locus and expected heterozygosity were calculated with Cervus [37]. Linkage disequilibrium (LD) between all loci pairs and deviations from the Hardy-Weinberg equilibrium (HWE) for all loci were calculated in Genepop [38]. To estimate genetic diversity for each microsatellite, Fis was calculated for every locus using Fstat [39].

#### MHC characterization within populations

Allele frequency, null allele frequency, observed heterozygosity (Ho) and expected heterozygosity (He) were calculated with Cervus [37]. Allelic richness (AR) and number of private alleles were calculated with GenAlEex [40]. Departures from the HWE were calculated with Genepop [38].

#### MHC differences within and between populations

The difference in allele frequencies among breeds was tested with the Chi-square test, and Bonferroni correction was applied to adjust the p-value for multiple comparisons. Tests were performed in PS IMAGO 5, IBM SPSS Statistics v.25 statistical package (IBM Corporation, Armonk, NY, USA).

Analysis of molecular variance (AMOVA) within and among populations and individuals was performed in GenAlEx [40].

#### Genetic structure

To perform genetic clustering of the individuals according to MHC microsatellites, the Bayesian model of population structure analysis was applied in Structure v2.3.4 software [41]. The following settings were applied: K = 2 to K = 10; 100 000 burns in; 200 000 Markov chain Monte Carlo (MCMC) iterations and 20 replicates. To determine the true value of K, the Evanno method was used [42]. Default settings were used (an admixture model with correlated frequencies) for all runs. AMOVA was performed across and within inferred clusters in GenAlEx [40].

#### Frequency of genetic events

The frequency of recombination and gene conversion was calculated based on the number of meiotic events, that is, the number of parent-offspring pairs, present in every population. In addition, the proportion of new haplotypes present in the offspring to the number of meiotic events was determined.

## Results

### MHC microsatellite occurrence

Amplified MHC microsatellites represented three different classes of equine MHC: MHC class I (MHC I), MHC class II (MHC II) and MHC class III (MHC III). A different number of alleles, ranging from five to fourteen, was detected for each locus. However, some alleles were rare and were detected in only a few individuals (S2 Table). Locus ABGe9019 (MHC III) was the most polymorphic, with Ho = 0.95 and 13 alleles, while locus UMN-JH38 (MHC I) was the least polymorphic, with Ho = 0.31 and 5 alleles. Fis values ranged from -0.07 (UM011) to 0.4 (TKY3324). Nine out of eleven loci showed a significant departure from the HWE. LD was detected between all pairs of loci. The results of MHC microsatellite analysis are shown in Table 1.

**Table 1:** Characteristics of MHC microsatellites.

Locus	N	Ho	He	Fis	HWE
COR110	12	0.72	0.87	0.17	***
TAMU30593	10	0.62	0.79	0.22	***
UMN-JH38	5	0.31	0.34	0.07	*
TKY3324	11	0.42	0.71	0.40	***
COR112	10	0.54	0.79	0.32	***
COR113	14	0.65	0.82	0.21	***
COR114	8	0.63	0.76	0.17	***
UM011	14	0.88	0.82	-0.07	***
ABGe9030	7	0.49	0.73	0.33	***
ABGe9019	13	0.95	0.89	-0.07	ns
UMNe65	10	0.79	0.85	0.07	ns

N, number of identified alleles; Ho, observed heterozygosity; He, expected heterozygosity; Fis, MHC microsatellite locus inbreeding coefficient; HWE, a departure from the Hardy-Weinberg equilibrium (* p < 0.05; *** p < 0.0001; ns, not significant)

### MHC characteristics within populations

Five microsatellites, namely, COR110, TAMU3059, UM011, ABGe9019 and UMNe65, were highly polymorphic (Ar, Ho, He, PIC) in all three populations, with the highest values of AR = 10.35; Ho = 0.96; He = 0.88 and PIC = 0.86 for ABGe9019 in Pop1. Similarly, high polymorphism was observed for COR114 in Pop2 and Pop3 and TKY3324 in Pop3. In contrast, UMN-JH38 was characterized by very low polymorphism, with PIC less than 0.5 in all three populations. The same was observed for COR112 and ABGe9030 in Pop3 (PIC<0.5). A high null allele frequency was detected for TKY3324 in Pop1 (0.14), Pop2 (0.42) and Pop3 (0.13); the TAMU30593 locus and COR112 in Pop2 (0.17 and 0.16, respectively); and the COR110 and TAMU30593 loci in Pop1 (0.14 and 0.11, respectively). Significant deviations from the HWE were observed in all populations; however, Pop1 and Pop2 had the highest number of loci that were not consistent with the HWE, that is, five and six loci, respectively. Detailed results are shown in Table 2.

**Table 2:** MHC characteristics within populations.

Population	MHC microsatellite	AR	Ho	He	PIC	Null allele frequency	HWE
**Pop1**	COR110	9.72	0.62	0.83	0.81	0.14	***
	TAMU30593	8.38	0.60	0.74	0.70	0.11	***
	UMN-JH38	2.0	0.33	0.33	0.31	0.02	ns
	TKY3324	5.79	0.38	0.53	0.50	0.14	***
	COR112	6.62	0.54	0.59	0.56	0.06	ns
	COR113	7.81	0.66	0.67	0.65	0.00	ns
	COR114	6.21	0.57	0.57	0.54	0.00	ns
	UM011	9.10	0.84	0.70	0.67	-0.13	*
	ABGe9030	4.98	0.47	0.54	0.50	0.06	**
	ABGe9019	10.35	0.96	0.88	0.86	-0.04	ns
	UMNe65	7.59	0.82	0.83	0.80	0.00	ns
**Pop2**	COR110	8.0	0.81	0.84	0.80	0.00	ns
	TAMU30593	7.0	0.53	0.72	0.67	0.17	***
	UMN-JH38	4.0	0.41	0.48	0.44	0.12	ns
	TKY3324	10.0	0.34	0.85	0.82	0.42	***
	COR112	5.0	0.50	0.68	0.63	0.16	*
	COR113	10.0	0.69	0.79	0.75	0.07	*
	COR114	6.0	0.72	0.77	0.73	0.03	ns
	UM011	8.0	1.0	0.84	0.80	-0.09	*
	ABGe9030	6.0	0.56	0.66	0.61	0.08	ns
	ABGe9019	8.0	0.94	0.80	0.76	-0.09	ns
	UMNe65	10.0	0.84	0.84	0.80	-0.02	*
**Pop3**	COR110	7.0	0.92	0.85	0.82	-0.04	ns
	TAMU30593	6.84	0.74	0.78	0.74	0.02	ns
	UMN-JH38	2.0	0.18	0.17	0.15	-0.04	ns
	TKY3324	8.66	0.61	0.80	0.76	0.13	**
	COR112	4.00	0.55	0.57	0.48	0.01	ns
	COR113	4.0	0.58	0.68	0.60	0.08	ns
	COR114	5.0	0.71	0.77	0.72	0.03	ns
	UM011	8.66	0.90	0.82	0.78	-0.05	ns
	ABGe9030	4.0	0.50	0.51	0.46	0.00	ns
	ABGe9019	8.95	0.92	0.85	0.82	-0.05	ns
	UMNe65	6.0	0.68	0.78	0.74	0.07	ns

AR, allelic richness; Ho, observed heterozygosity; He, expected heterozygosity; PIC, polymorphism information content; HWE, a departure from the Hardy-Weinberg equilibrium (* p < 0.05; *** p < 0.0001; ns, not significant). Pop1, Polish draft horses; Pop2, semiferal Konik Polski horses; Pop3, Konik Polski horses, stabled group

### Allelic pattern of MHC loci and genetic differences within MHC among populations

Allele frequencies differed between populations in all loci (p<0.0001). The highest number of private alleles was detected in Pop1 (2.09), while the highest overall heterozygosity was observed in Pop2 (He = 0.74) (Table 3).

**Table 3:** Allelic pattern of MHC loci among populations (Pop1, Pop2, Pop3).

Population	Na	Ne	Np	He
Pop1	8.36	3.65	2.09	0.65
Pop2	7.27	4.33	0.64	0.74
Pop3	6.0	3.98	0.36	0.68

Na, number of different alleles; Ne, number of effective alleles; Np, number of private alleles; He, expected heterozygosity calculated for a population. Pop1, Polish draft horses; Pop2, semiferal Konik Polski horses; Pop3, Konik Polski horses, stabled group

**Fig 1:**
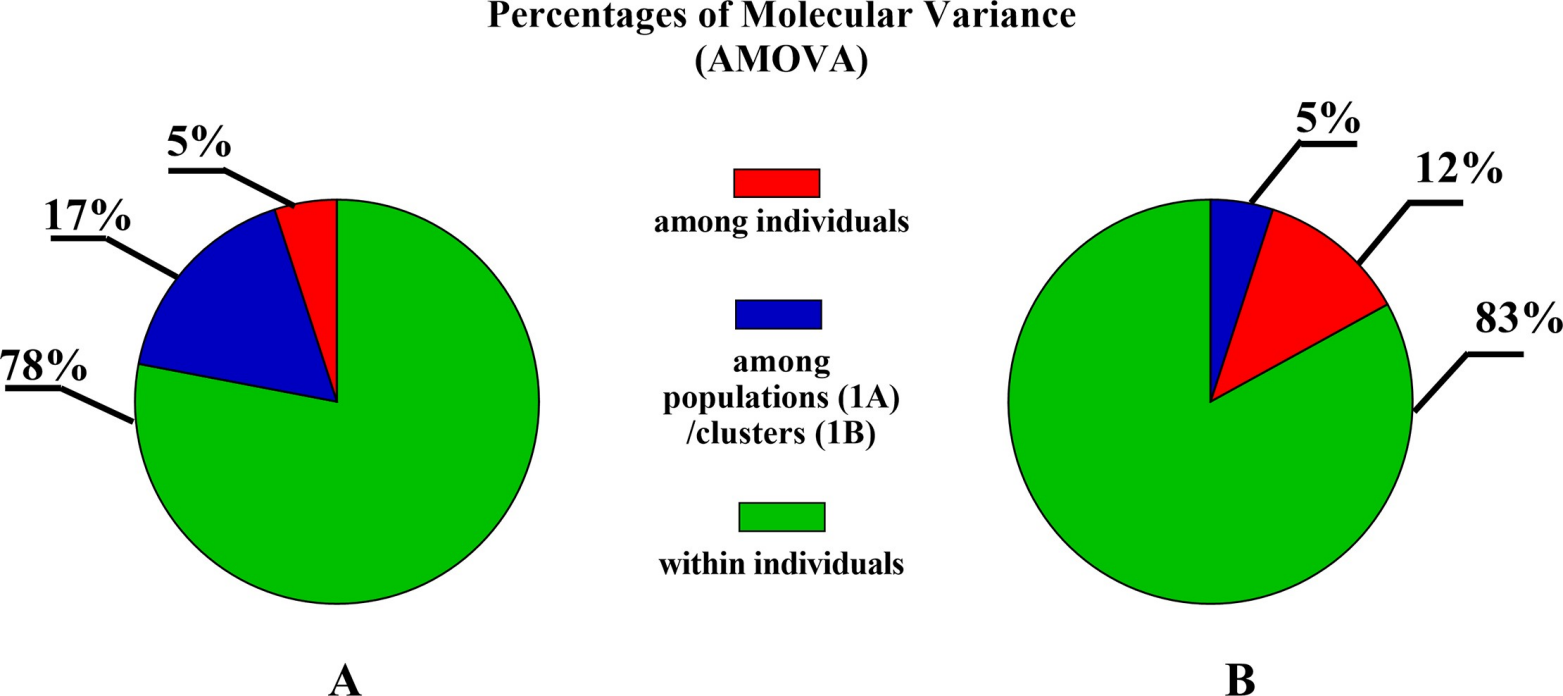
Genetic differentiation among populations (Pop1, Pop2, Pop3; Fig 1A) and clusters (C1, C2, C3, C4; Fig 1B) and among and within individuals calculated by AMOVA. Pop1, Polish draft horses; Pop2, semiferal Polish Konik horses; Pop3, Polish Konik horses, stabled group. Genetic clustering was based on the Bayesian method for K = 4.

The genetic differentiation among populations was 17% (Fig 1A). The most closely related populations (Fst = 0.02) were Pop2 and Pop3, while Pop1 and Pop3 were the most different (Fst = 0.11). Fst = 0.1 was observed between Pop1 and Pop2. Similar results were obtained when unbiased Nei genetic distance (Nei D) and genetic identity (Nei I) were analyzed. Pop2 and Pop3 were very similar genetically (Nei D = 0.07; Nei I = 0.93), and Pop1 and Pop3 were the most distinct (Nei D = 0.71; Nei I = 0.49). The genetic distance between Pop1 and Pop2 based on the Nei indices was Nei D = 0.66; Nei I = 0.52.

### Structure results

**Fig 2:**
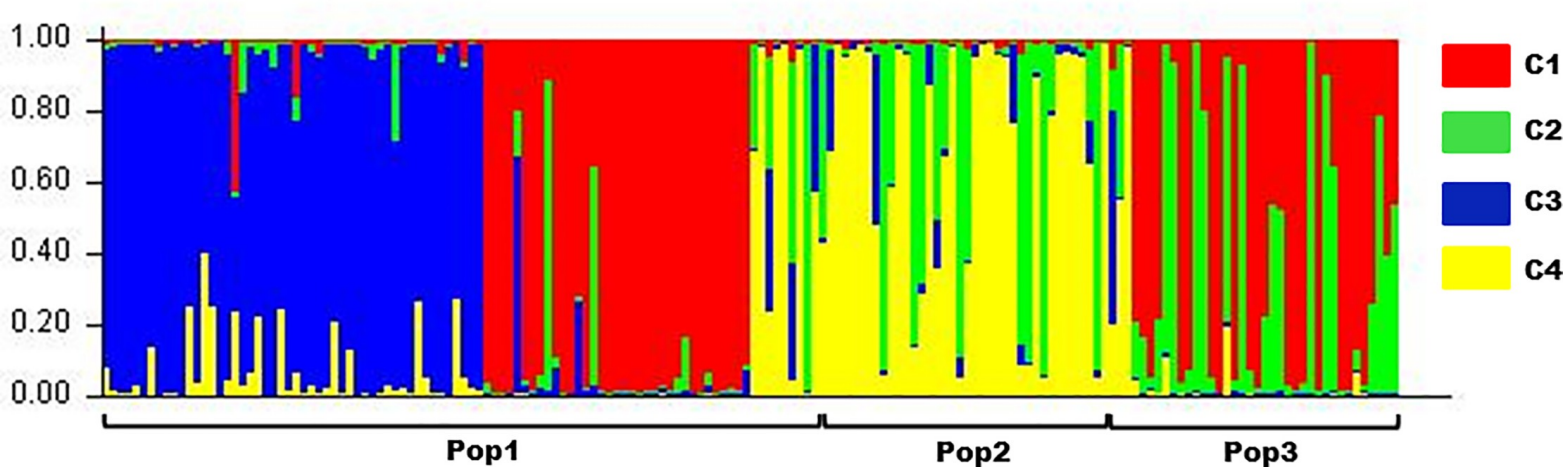
Genetic clustering of horses from all populations (Pop1, Pop2, Pop3) based on the Bayesian model for K = 4. Pop1, Polish draft horses; Pop2, semiferal Konik Polski horses; Pop3, Konik Polski horses, stabled group. The colors apply to different genetic clusters (C1, C2, C3, C4).

Based on the applied method [41], the number of clusters in the final analysis was set to K = 4 (S1 Fig). Pop1, which consisted of Polish draft horses, was separated among all 4 genetic clusters (C1, C2, C3, C4); however, a majority of the horses were distributed between two clusters (C1, C3). Within Pop1, 91% of the horses showed a greater than 70% assignment rate to a cluster. Pop2, consisting of semiferal Polish Konik horses, was separated between two clusters (C2, C4), and 78% of these horses had an assignment rate of 70% or higher to one of the clusters. Polish Konik horses from the stabled group, similar to the draft horses. were divided among all four clusters; nevertheless, a majority fit in two clusters (C1, C2), with 82% of horses having an assignment rate above 70%. The results of clustering are shown in Fig 2.

The genetic differentiation between clusters was 5% (Fig 1B). In general, clustering based on MHC microsatellite alleles decreased the genetic distance between newly assigned groups of horses. Differences were detected between C2 and C3 (Fst = 0.08; Nei D = 0.5; Nei = 0.6), while the other possible cluster combinations were almost identical genetically (Fst = 0.01 to Fst = 0.04; Nei D = 0.04 to Nei D = 0.2; Nei I = 0.8 to Nei I = 1).

### MHC haplotypes

In Pop1, consisting of Polish draft horses, 111 haplotypes were identified. None of these haplotypes were present in any of the populations of Konik Polski horses. In Pop2, 43 MHC haplotypes were identified in the semiferal Konik Polski horses, whereas 49 haplotypes were identified in Pop3, consisting of stabled Konik Polski horses. Four of these haplotypes were present in both populations of Konik horses (Pop2 and Pop3).

### Recombination frequency

We detected 15 recombination events; six recombinations occurred in Pop1, three in Pop2 and two in Pop3:

Polish draft horses:

- Between ABGe9019 and UMNe65 (MHC III); horse 26
- Between UM011 and COR114 (MHC II) and between UMNe65 and ABGe9030 (MHC III/MHC II); horse 74
- Between COR112 and COR113 (MHC II) and between UM011 and COR114 (MHC II); horse 44
- Between UMNe65 and ABGe9030 (MHC III/MHC II) and between COR113 and UM011(MHC II); horse 52
- Between UMNe65 and ABGe9030 (MHC III/MHC II) and between UM011 and COR114 (MHC II); horse 78
- Between ABGe9019 and UMNe65 (MHC III), between TKY3324 and COR112 (MHC II), and between UM011 and COR114 (MHC II); horse 12

Semiferal Konik Polski horses:

- Between TAMU30593 and ABGe9019 (MHC I/MHC III); horse 102
- Between ABGe9019 and UMNe65 and between ABGe9030 and TKY3324 (MHC III/ MHC II); horse 104
- Between TAMU30593 and ABGe9019 (MHC I/MHC III), between TKY3324 and COR112 (MHC II) and between COR113 and UM011 (MHC II); horse 112

Stabled Konik Polski horses:

- Between ABGe9019 and UMNe65 (MHC III), between COR112 and COR113 (MHC II), and between UM011 and COR114 (MHC II); horse 164
- Between UM011 and COR114 (MHC II); horse 148

The highest recombination frequency (12.5%) was detected in Pop2. The recombination frequencies in Pop1 and Pop3 were similar (7% and 6.7%, respectively).

**Fig 3:**
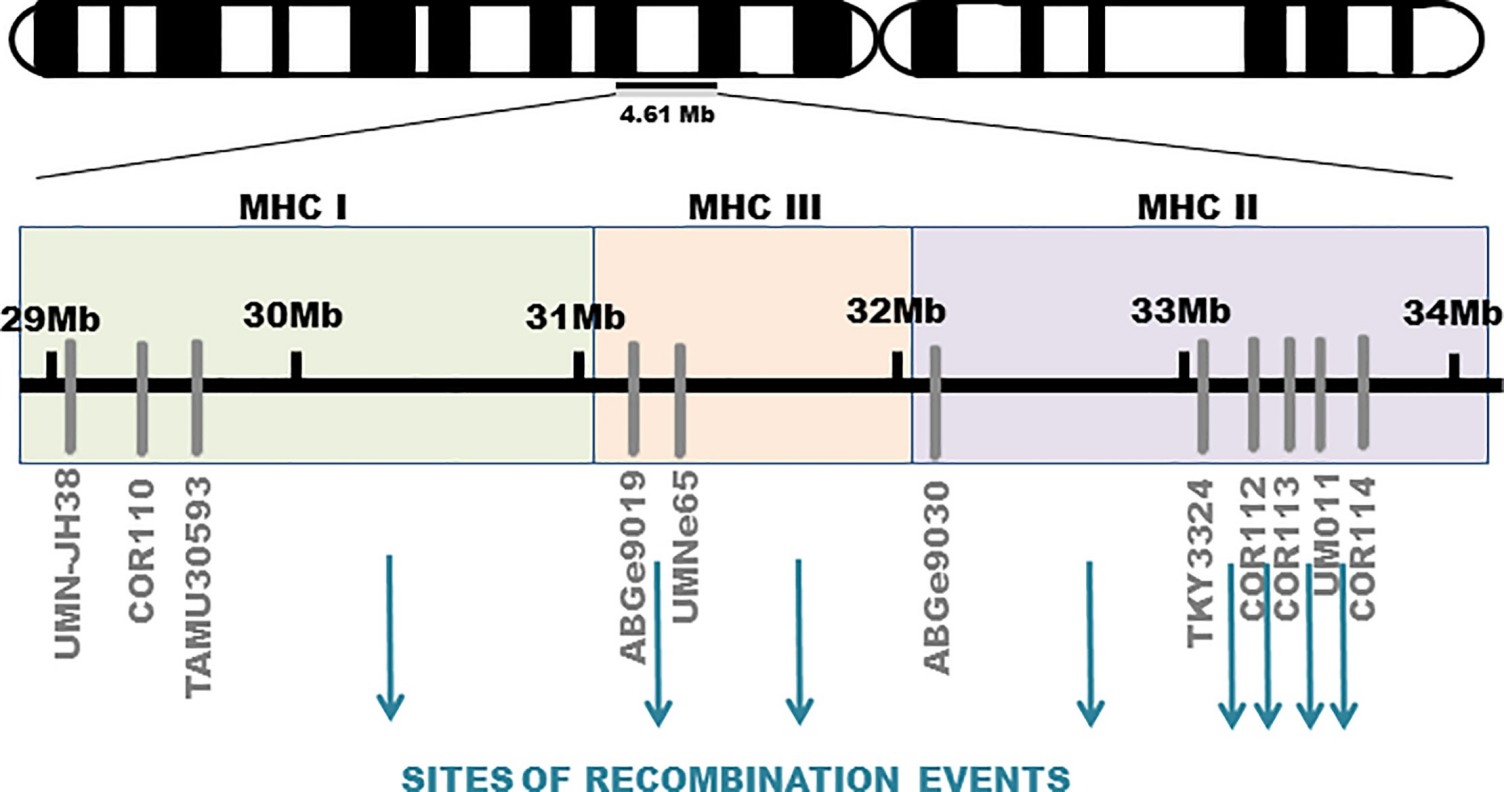
Map of intra-MHC microsatellites used in the study. MHC class I, MHC class II and MHC class III regions are located on the equine chromosome 20 and highlighted. Arrows indicate sites of recombination events within the equine MHC region in the studied population of horses.

A majority of the recombination events occurred in the MHC class II and class III regions (Fig 3). In every case, the recombinant haplotype was of paternal origin.

**Fig 4:**
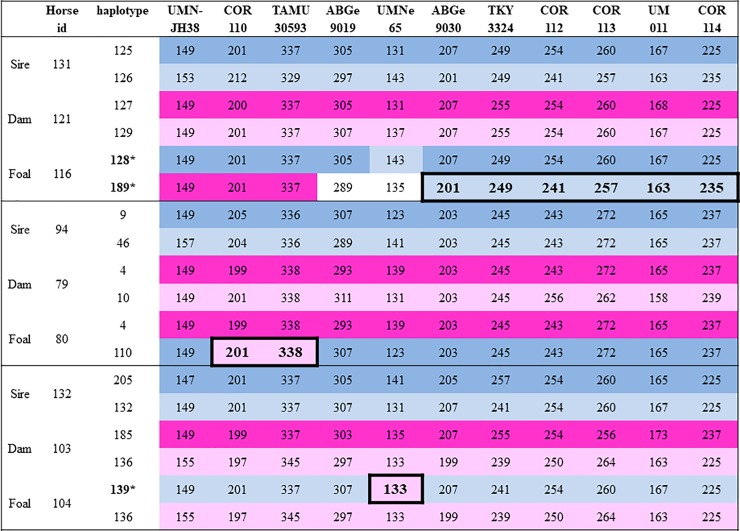
Events of intra-MHC conversion present in the offspring (foal 116, foal 80, foal 104). Fragments of haplotypes which were converted are marked. Every conversion resulted in new haplotype (*).

Interestingly, we detected gene conversion in the MHC haplotype of two fillies from Pop2. Conversion occurred in the MHC III region (Fig 4; horse 104, horse 116). In Pop1, there was one gene conversion in the MHC I region, and this filly inherited both alleles in the COR110 and TAMU30593 loci from the dam (Fig 4, horse 80). The gene conversion frequency was 4% in Pop2 and 1.2% in Pop1. No conversion events were detected in Pop3.

In addition, mutations such as insertions (20 events) or deletions (seven events) in single MHC loci were detected in all three populations. None of these mutations resulted in the new allele in any of these populations.

As a result of all of the genetic events, seven new haplotypes emerged in Pop1, eleven in Pop2 and eleven in Pop3. The ratio of new haplotypes to the number of meiotic events was highest in Pop2 (46%), followed by Pop3 (37%) and Pop1 (8%).

## Discussion

In humans and animals, microsatellites are conventionally used for genome characterization, identification of species and individuals, parentage verification, pedigree analysis, and testing for genetic disorders and as markers for phenotypic traits [43–45]. According to the recommendation of the International Society for Animal Genetics (ISAG), STRs (short tandem repeats) are routinely used in parentage testing in numerous species, including horses [46–49]. In addition to sequencing, microsatellites are useful for investigating the evolution of genes, including MHC [50–52]. The presence or absence of specific microsatellite alleles is associated with the occurrence of some diseases in both animals and humans [25, 28, 53–56]. Therefore, in the present report, the panel of microsatellites was applied to evaluate the diversity of MHC within and between populations of native and draft horses. Microsatellites allow evaluation of diversity in not only a single locus but also the haplotype, that is, a set of alleles present in an individual [33], which makes this assessment highly accurate and reliable. In horses, similar to other species, MHC microsatellites are reported to be in high linkage disequilibrium with MHC genes [33]. Thus, the polymorphism observed in these genetic markers should reflect polymorphisms in MHC genes of investigated horses.

In agreement with other studies, loci of MHC II and MHC III showed the highest polymorphism in the studied populations of horses. In both populations of Konik Polski horses, the number of highly polymorphic MHC microsattelite loci (PIC>0.6) was higher than that in Polish draft horses (Table 2). A similar level of polymorphism was reported for the COR110, COR112, COR113, COR114, ABGe9019, ABGe9030, UMO11, and UMNe65 loci in Arabian horses [35].

Considering the location of the microsatellites in the chromosome and their correspondence to different MHC classes, the highest polymorphism was detected in MHC III microsattelites. In horses, MHC III genes are poorly characterized, although it is believed that, similar to humans [57], these genes encode heat shock proteins, complement components and cytokines, such as those of the tumor necrosis factor family [29]. Thus, the products of this MHC region play a central role in innate immune defense [57]. In humans, diversity in the genes encoding C4, one of the components of the complement, is suggested to be an evolutionary adaptation of the innate immune system to a wide range of parasites [58]. Similar mechanisms may be present in horses. Moreover, it has been suggested that specific MHC III alleles are associated with some autoimmune and infectious diseases in humans [57, 59]. Whether such associations are present in horses remains to be determined.

Interestingly, among MHC I microsatellites, the UMNJH-38 locus showed the lowest polymorphism in all studied populations (Konik Polski and Polish draft horses), which is consistent with the results for UMNJH-38 in Arabian horses [35]. Despite the low polymorphism of this marker, the allelic range of this locus differs between Konik Polski horses (147–155), draft horses (149–157), Arabian horse (156–165) [35], Icelandic horses (156–165) [36] and other breeds tested (thoroughbreds, standardbreds and quarter horses (156–163) [32], suggesting breed-related differences in UMNJH-38, although this marker needs to be studied in more breeds to validate this statement. In addition, differences in MHC I-encoded proteins are associated with susceptibility to equine herpes virus type 1 and 4 (EHV1, EHV4) infection. The abovementioned horse breeds have different histories of breeding and exposure to environment-specific pathogens, including herpes viruses [26, 27]. Considering our results, a question arises as to whether disparity in the susceptibility to common pathogens might also be related to breeding.

Unexpectedly, both populations of Konik Polski horses had higher genetic diversity than draft horses. The breeding policy for both breeds varies: Polish draft horses, unlike Koniks, have an open studbook, and admixture of other draft breeds is a part of the breeding strategy [60]. In general, the MHC diversity of Polish draft horses and Konik Polski horses corresponds to the diversity assessed by neutral markers reported in domestic horses, which ranges between 0.43 and 0.79 [15, 61, 62]. Nevertheless, a study on the genetic structure of Polish draft horses reported diversity within neutral markers at the level of 0.39 [15]. In contrast, studies on the genetic variability of Konik Polski horses showed values of He equal to 0.86 [20] and 0.7 [21]. Polish draft horses are currently bred mainly for meat; thus, individuals with good musculature are valued. Although the admixture of other draft breeds is usually allowed, only horses with the desired phenotype are left for breeding and enter the studbook [15, 60]. Additionally, draft stallions bred 16–17 mares a year (https://www.pzhk.pl/), and highly valued stallions bred up to 700 mares during their breeding career [60]. Thus, it can be inferred that the genetic contribution in the breed of highly valued horses, especially stallions, is high in Polish draft horses. In conclusion, this breeding strategy may lower the genetic diversity in this breed. Nevertheless, our results are similar to those obtained in other draft horses [61, 63]. In contrast, Konik Polski horses are kept as a pure breed. Due to the conservation program, in the conventional breeding system, matings between mares and stallions are carefully planned. In feral conditions, mating is not controlled by humans and occurs mostly between harem stallions and their mares. Nevertheless, according to the Konik Polski studbook, every stallion, regardless of the type of maintenance (conventional or feral), breeds seven to ten mares a year (https://www.pzhk.pl/). Thus, in Polish draft breeds, few stallions contribute to the population gene pool, whereas in Koniks, almost every stallion enters the studbook. Interestingly, Konik Polski horses are characterized by their vitality and pathogen resistance, which could be associated with their MHC genes [19, 20, 64]. Moreover, environmental factors such as feral conditions in the maintenance of semiferal Konik Polski horses might influence the diversity within the MHC region. This speculation is further confirmed by studies in which semiferal Konik Polski horses were more resistant to pathogens than stabled Koniks [65].

The breeding history of Polish draft horses and Konik Polski horses was separate for approximately 70 years. Similarly, the semiferal and stabled populations of Konik horses from our study were not crossbred since that time. The allele frequencies are significantly different among all three populations in every microsatellite locus. Specific MHC microsatellite alleles are associated with autoimmune-based diseases such as insect bite hypersensitivity or recurrent uveitis in some horse breeds [24, 25]. The predisposition to certain diseases clearly differs between Polish draft horses and Konik horses. Polish draft horses, similar to Friesians, suffer from a high incidence of retained fetal membranes [66–68]. In Friesians, cows, and humans, this disease is suggested to be genetically inherited; however, no association with specific genes or gene markers has been discovered to date [17, 69, 70]. On the other hand, owners of stabled Konik horses claim a high frequency of recurrent airway obstruction; however, no studies on large cohorts of horses have been performed. It can be speculated that susceptibility to some diseases present in some horse breeds and not in others might be associated with allele segregation caused in part by artificial selection.

The obtained results showed close genetic distance between Konik Polski horses and Polish draft horses, which could be expected considering the past cross-breeding between these breeds. Interestingly, the few Polish draft horses that were not assigned to clusters grouping this breed were the breeding stallions. These horses were clustered with Konik Polski horses. These results may support two hypotheses. First, neither of the breeds is yet differentiated, which confirms the common breeding history. Second, MHC genes are inseparably connected with the fitness of an individual. Studies on diversity based on neutral markers and/or MHC show inconsistent results [8, 71, 72]; the diversity in MHC is either higher [73, 74] or lower [75, 76] than that in the rest of the genome. It can be speculated that in addition to the pressure of artificial selection and breeding for desired traits, as in the horse industry, the diversity within MHC genes in domestic horses is influenced by the pathogen load present in a certain environment, which can differ from population to population even within the same breed. Furthermore, the pool of functional genes, such as MHC, even in domestic horses undergoing artificial selection, might still depend on environmental factors. A study by Stachurska et al. [77] on genetic distances between common breeds in Poland estimated the Nei genetic distance between Konik polski and Polish draft horses based on erythrocyte antigen and protein loci as 0.04, which is less than any of the Nei’s distances calculated between the populations used in the present study (0.07 to 0.71). When genetic clustering was applied, the differentiation between horses based on MHC microsatellite alleles decreased (0.04 to 0.6); however, the clusters were still more genetically distinct than when neutral markers were used in the calculation. To our knowledge, no other studies have estimated the genetic distance between Polish Konik and Polish draft horses. These differences in genetic distance between Polish Konik and Polish draft horses, depending on the markers used, may further confirm the influence of the environment on MHC diversity in domestic horses.

All three populations showed high diversity of MHC haplotypes. Almost the same panel of microsattelites was used in Arabian horses [35], Icelandic horses [36] and other horse breeds [32]; thus, we were able to compare the results. None of the haplotypes present in Konik Polski and Polish draft horses were present in the other previously studied breeds. Moreover, none of the Polish draft horse haplotypes were present in populations of Koniks, and even in this breed, the MHC haplotypes differed. We speculate, similar to Holmes et al. [36], that both breed separation and the limited number of breeds investigated for their MHC haplotypes are reasons for the uniqueness of the obtained haplotypes. Nevertheless, we think that this result also highlights the polymorphisms present in equine MHC.

Enrollment of family trios and parent-offspring pairs of horses allowed us to show possible mechanisms underlying MHC haplotype diversity in horses [35, 36]. The MHC III region has been proposed as a recombination hotspot for equine MHC haplotypes [35, 36], which is consistent with our results. Indeed, approximately half of the observed recombination events occurred in MHC III or between MHC III and MHC II. However, based on our results, MHC II might be suggested as another recombination hotspot. The MHC II region has been proposed as the recombination hotspot in bovines [78], other ungulates [79] and humans [80]. As in previous reports [35, 36], new haplotypes were generated mostly by recombination.

Notably, the recombination rate in semiferal Konik Polski horses was higher than those in the other two populations. Unlike other horses that undergo procedures such as deworming or any necessary medical interventions, semiferal Konik horses do not. Pathogen pressure is believed to be one of the major causes of MHC polymorphisms [81]. Parasites and other pathogen species can change over time; thus, different MHC alleles or allelic combinations may be favored. This continuous pathogen-host race may be observed as early as in the next generation of sensitive species [81] by tracking changes in both the MHC alleles and MHC haplotypes. Nevertheless, MHC is a family of genes; thus, its functions, such as immune defense, often depend on the changes in the combination of a whole set of alleles, that is, the MHC haplotype [58]. It can be speculated that, in comparison to the recombination rate in stable-maintained horses, the recombination rate in semiferal horses is the adaptation of a differently challenged immune system to changing pathogens. On the other hand, the recombination frequency in humans varies depending on the population [80]. In our studies, three isolated populations of horses were used. None of these hypotheses is mutually exclusive because every population lives in a distinctive environment inhabited by different pathogens. Moreover, the MHC polymorphism calculated for single loci was similar across all three tested populations. However, the proportion of new haplotypes was the highest in the offspring from semiferal Konik Polski horses.

Gene conversion is one of the mechanisms underlying MHC diversity across species [82]. By definition, gene conversion is a “unidirectional exchange of the genetic material between homologous sequences of single or multiple loci” [82]. It is suggested that this mechanism is responsible for high genetic variation in the human MHC II region. Gene conversion has also been proposed as one of the mechanisms of MHC haplotype variability in small populations where migration is limited [43]. We suspect that intra-MHC conversion was detected in Pop1 in MHC I and Pop2 in MHC II and MHC III. Pop2 is relatively small, and mares breed with the same stallion for many consecutive years. As in other species, intra-MHC conversion may be an additional mechanism, next to recombination, to maintain MHC-based pathogen resistance in small, isolated populations [83].

Unlike intra-MHC recombination and conversion, insertion and deletion did not result in the generation of new alleles; however, some of these events resulted in the diversification of new haplotypes. Thus, it can be speculated that in horses, these events, together with recombination and conversion, contribute to MHC diversity.

## Conclusion

Breeding for selective traits, as in the horse industry, may decrease genetic diversity in both breeds and individuals. With regard to functional genes, such as MHC, such breeding may negatively influence individual fitness, which may decrease the value of an animal. In Konik Polski and Polish draft horses, MHC diversity is similarly high despite the different breeding policies. However, semiferal Konik Polski horses experienced a higher number of MHC gene recombination and conversion events than the population of stabled horses of the same breed or Polish draft horses, which resulted in a higher proportion of new MHC haplotypes in the offspring. Thus, we speculate that the genetic makeup of the horse MHC might be more strongly influenced by the environment than by artificial selection.

## Supporting information

S1 Fig Estimated value of K = 4 calculated from the results from the initial population structure analysis by the Evanno method. The following settings were applied: K = 2 to K = 10; 100 000 burns in; 200 000 Markov chain Monte Carlo (MCMC) iterations and 20 replicates.
(TIF)

S1 Table Primers for eleven MHC microsatellites. Sizes of the amplicons and gene accession numbers and/or references are provided where applicable.
(DOCX)

S2 Table MHC microsatellite alleles and haplotypes found in the studied population. (XLSX)
