# Supplementary figures and images for: Inter- and intrabreed diversity of the major histocompatibility complex (MHC) in primitive and draft horse breeds
Source: PLoS One. 2020 Feb 3;15(2):e0228658. doi: 10.1371/journal.pone.0228658 (PMC6996847; doi:10.1371/journal.pone.0228658)

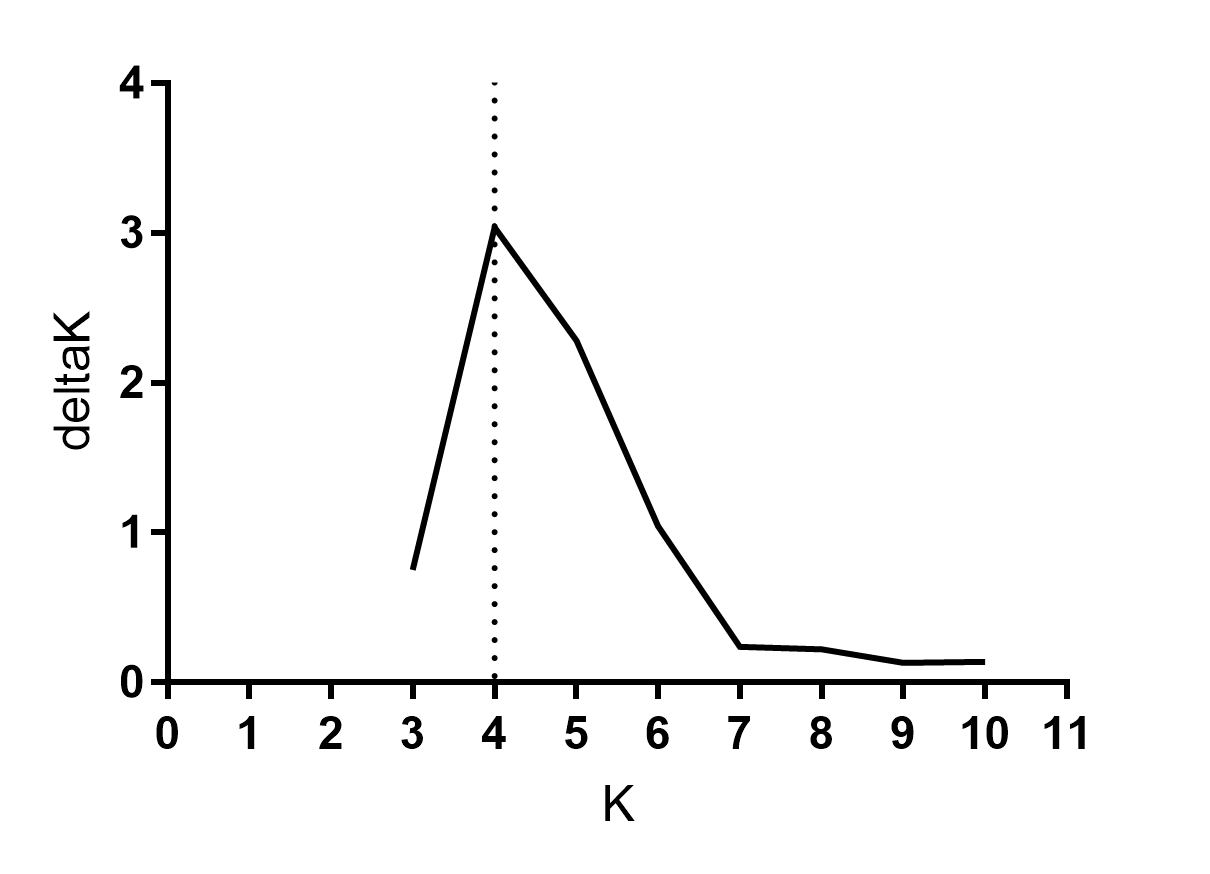

Supplement: S1 Fig — The following settings were applied: K = 2 to K = 10; 100 000 burns in; 200 000 Markov chain Monte Carlo (MCMC) iterations and 20 replicates. (TIF) [file pone.0228658.s001.tif]
