# Supplementary material for: Inter- and intrabreed diversity of the major histocompatibility complex (MHC) in primitive and draft horse breeds
Source: PLoS One. 2020 Feb 3;15(2):e0228658. doi: 10.1371/journal.pone.0228658 (PMC6996847; doi:10.1371/journal.pone.0228658)
Supplement: S1 Table — Sizes of the amplicons and gene accession numbers and/or references are provided where applicable. (DOCX) [file pone.0228658.s002.docx]

**S1 Table. Primers for eleven MHC microsatellites sizes of the amplicons and gene accession numbers and/or references where applicable.**

|  | **microsatellite** | **MHC Class region** | **primer** | **sequence** | **obtained product size** | **label** | **Gene bank accession number/ reference** |
| --- | --- | --- | --- | --- | --- | --- | --- |
| MULTIPLEX 1 | **ABGe9019** | III | forward reverse | CTGAGAGAGACAGCATTTGTGG GAAAGGTGTCTCCATTCTTGCT | 291-311 | NED | FN414912.1 |
|  | **ABGe9030** | II | forward reverse | CCAGCAGACCTGCAAGAGTA  AGCATGAGAGCCATGAAGGT | 193-209 | NED | FN414920.1 |
|  | **TKY3324** | II | forward reverse | AGCCGTCCTGTTCCAACTAA  TGCCCCTTAAAACTCTGTCTTT | 239-261 | NED | AB217267.1 |
|  | **COR110** | I | forward reverse | TTTGGTCTTTGCAGGTATGG  TCTCCCTTCCTCTTTGTTCC | 197-214 | FAM | EF531702 |
| MULTIPLEX 2 | **COR112** | II | forward reverse | TTACCTGGTTATTGGTTATTTGG  TCACCCACTAAATCTCAAATCC | 230-260 | NED | [32] |
|  | **TAMU30593** | I | forward reverse | GAAGCCCAGTCTGAGTGAAGAT AGATTTGGACCGAGAAAGTCTG | 329-345 | FAM | [36] |
| MULTIPLEX 3 | **COR113** | II | forward reverse | TGTTTAGAACTCGCCAGGAG  TCATCAGTTCCTTGCCTAGC | 248-272 | NED | [32] |
|  | **COR114** | II | forward reverse | TCAAAATCCACACTCCCTTC  TCCATAAAGAGTGGGACACTG | 225-245 | FAM | [32] |
|  | **UM011** | II | forward reverse | TGAAAGTAGAAAGGGATGTGG  TCTCAGAGCAGAAGTCCCTG | 158-173 | NED | AF195130 |
|  | **UMN-JH38** | I | forward reverse | TGTGTGTGCACCTGTCCTTT  GATGGGAGGGAATGAGGAAT | 149-157 | FAM | EF531700.1 |
|  | **UMNe65** | III | forward reverse | TCCTTCCACTCCCCTCAAC TCCCTGAAAAACCTTGGTTG | 115-143 | FAM | AF191698.1 |
